# Cortical functional correlates of responsiveness to short-lasting preventive intervention with ketogenic diet in migraine: a multimodal evoked potentials study

**DOI:** 10.1186/s10194-016-0650-9

**Published:** 2016-05-31

**Authors:** Cherubino Di Lorenzo, Gianluca Coppola, Martina Bracaglia, Davide Di Lenola, Maurizio Evangelista, Giulio Sirianni, Paolo Rossi, Giorgio Di Lorenzo, Mariano Serrao, Vincenzo Parisi, Francesco Pierelli

**Affiliations:** Don Carlo Gnocchi Onlus Foundation, Milan, Italy; Department of Neurophysiology of Vision and Neurophthalmology, G. B. Bietti Foundation-IRCCS, Rome, Italy; Department of Medico-Surgical Sciences and Biotechnologies, “Sapienza” University of Rome Polo Pontino, Latina, Italy; Istituto di Anestesiologia, Rianimazione e Terapia del Dolore, Università Cattolica del Sacro Cuore/CIC, Rome, Italy; Delle Medical Center, Wellness and Dietary Medicine, Rome, Italy; INI, Headache Clinic, Grottaferrata, (RM) Italy; Department of Systems Medicine, University of Rome “Tor Vergata”, Laboratory of Psychophysiology, Rome, Italy; INM Neuromed IRCCS, Pozzilli, (IS) Italy

**Keywords:** Ketogenesis, Diet, Habituation, Migraine, Visual-evoked potentials, Somatosensory-evoked potentials

## Abstract

**Background:**

Here, we aim to identify cortical electrofunctional correlates of responsiveness to short-lasting preventiveintervention with ketogenic diet (KD) in migraine.

**Methods:**

Eighteen interictal migraineurs underwent visual (VEPs) and median nerve somatosensory (SSEPs) evokedpotentials before and after 1 month of KD during ketogenesis. We measured VEPs N1-P1 and SSEPs N20-P25 amplitudes respectively in six and in two sequential blocks of 100 sweeps as well as habituation as theslope of the linear regression between block 1 to 6 for VEPs or between 1 to 2 for SSEPs.

**Results:**

After 1-month of KD, a significant reduction in the mean attack frequency and duration was observed (all *P*< 0.001). The KD did not change the 1st SSEP and VEP block of responses, but significantly inducednormalization of the interictally reduced VEPs and SSEPs (all *p* < 0.01) habituation during the subsequentblocks.

**Conclusions:**

KD could restore normal EPs habituation curves during stimulus repetition without significantly changing theearly amplitude responses. Thus, we hypothesize that KD acts on habituation regulating the balancebetween excitation and inhibition at the cortical level.

## Background

Migraine accounts for the highest headache-related disability of the primary headaches. Despite the great knowledge achieved on the pathophysiology of migraine, commonly available preventive treatments are effective in only about half of patients. Many have disabling and uncomfortable adverse effects. Pharmacological and non-pharmacological treatments that are more effective are very welcome [1–3]. In recent decades, specific dietetic interventions raised the interest of experts in the field of headaches. The evidence for an association between migraine, obesity [4, 5], and metabolic syndromes such as insulin resistance [6, 7] has supported this approach because they could be improved via specific dietary patterns.

We reported a case of a pair of overweighed twin sisters whose high-frequency migraine improved during a ketogenic diet (KD) that they followed to lose weight [8]. Their KD consists of a very low-calorie ketogenic diet (VLCKD) including a drastic restriction in carbohydrate and lipid intake that promotes fat metabolism and ketone body synthesis. After our initial accidental observation, we proved the clinical efficacy of VLCKD in a population of overweighed migraine patients. This was not related to diet-induced weight loss because the same improvement was not observed during a standard dietetic regimen [9]. According to the experimental observations in animal models, the underlying mechanisms of KD efficacy could be related to its ability to modulate cortical excitability [10, 11].

Time-locked cortical potentials evoked by a sensorial stimulation have been used frequently as a model to study migraine brain excitability. With these methods of clinical neurophysiology, researchers frequently observed a lack of cortical response habituation during any kind of stimulus repetition including visual and somatosensory [12, 13]. Habituation is a form of plastic learning and is a protective mechanism intended to limit cortical excitability. It prevents neuronal stress and excessive accumulation of metabolites such as lactate [14].

Here, in order to investigate if KD may exert its prophylactic effect regulating abnormal cortical information processing and excitability, we evaluated for the first time the influence of 1-month KD on habituation of visual and somatosensory cortical evoked potentials in a group of episodic migraineurs during the interictal phase.

## Methods

### Subjects

We initially enrolled 25 consecutive migraine patients who attended our headache centre. We discarded the recordings of seven patients who did not fulfil our primary inclusion criteria (see below). The final analysis set thus comprises a group of 18 migraine patients (14 without aura [MO, ICHD-III code 1.1], and four with aura [MA, ICHD-III code 1.2.1]) aged 19–54 years (mean 38.8 years). We took information on various clinical characteristics by collecting up to two-month headache diaries at the time of the screening visit. Patients had to indicate the duration of migraine history (years), attack frequency (n/month), and attack duration (hours). Of the enrolled migraineurs, we identified ten overweight patients (BMI ≥ 25, mean 28.6) and eight normal-weight patients (BMI < 25). For comparison, we included a group of 18 age- and gender- matched (one by one) healthy volunteers (HV, 16 women, mean age 38.8 years), with comparable BMI, we previously recruited among medical school students and healthcare professionals, who underwent both VEP and SSEP recordings.

### Ketogenic diet

Overweight patients received a 4-week low-carbohydrate (about 30 g/day carbohydrates), low-fat (fixed 15 g lipids), and normal protein (1.0–1.2 g/Kg of desired weight proteins) VLCKD diet (≤800 kcal) according to methods described elsewhere [8, 9]. This comprised five daily meals consisting of four protein shakes developed *ad hoc* as a protein supplement in KD (Ketoneural Protein Blend, Medi-Diet s.r.l., Aprilia, Italy), one daily meal of meat (up to 200 g) or fish (up to 350 g) accompanied by salad and nutraceutical integrators (Table 1). Further optional meals were allowed for non-sedentary subjects. Normal weight patients (BMI < 25) were administered a 4-week normo-caloric ketogenic dietary regimen (modified Atkins diet (MAD)) [15] consisting of low carbohydrate (about 15 g/day), normal/low protein (about 0.7 g/Kg/day, or less) and high fat (approximately little more than the weight of carbohydrates and proteins together) from meals prepared by common foods. These were supplemented with lipids in the form of a powder composed of medium chain triglycerides as well as omega-3 and long chain triglycerides (Ketoneural LipidiComplex, Medi-Diet s.r.l., Aprilia, Italy) with nutraceutical integrators (Table 1). For both groups, a daily urine dip stick test confirmed the presence of ketogenesis. Patients reported the stick results in a headache diary along with meals, daily weight, possible adverse events, or side effects. Patients had medical supervision and laboratory blood tests (alanine aminotransferase, aspartate aminotransferase, gamma glutamic transpeptidase, lactic dehydrogenase, alkaline phosphatase, bilirubin, blood urea nitrogen, and creatinine) at the start and the end of the 4-week KD.

**Table 1:** Nutraceutical integrators daily supplemented by patients during ketogenic diet, with doses expressed in milligrams (mg) and percentage of recommended daily allowance (RDA)

	daily dose	RDA
Vit A	800 mcg	100 % RDA
Vit B1	1.4 mg	100 % RDA
Vit B2	1.6 mg	100 % RDA
Vit B3	18 mg	100 % RDA
Vit B5	6 mg	100 % RDA
Vit B6	2 mg	100 % RDA
Vit B8	150 mcg	100 % RDA
Vit B9	200 mcg	100 % RDA
Vit B12	1 mcg	100 % RDA
Vit C	60 mg	100 % RDA
Vit D	5 mcg	100 % RDA
Vit E	10 mg	100 % RDA
Ca	800 mg	100 % RDA
Cr	7.5 mcg	15 % RDA
Cu	0.6 mg	50 % RDA
Mg	90 mg	30 % RDA
Mn	1.75 mg	RDA 1–10 mg
Zn	7.5 mg	50 % RDA

The primary inclusion criterion was being attack-free for at least 3 days before and after the recording sessions as determined by collecting headache diaries and telephone or e-mail interviews. Exclusion criteria were regular medication intake (i.e. antibiotics, corticosteroids, antidepressants, benzodiazepines, prophylactic migraine drugs) except for contraceptive pills. Other exclusion criteria included failure to reach a best-corrected visual acuity of > 8/10, history of other neurological diseases, systemic hypertension, diabetes or other metabolic disorders, connective or autoimmune diseases, and any other type of primary or secondary headache. Female participants were always recorded mid-cycle. All study participants were naïve to the study procedure and received a complete description of the study and gave informed consent. The project was approved by the Ethics Committee of the “Sapienza” University of Rome, Polo Pontino.

### Data acquisition

#### Visual-evoked potentials

Subjects were seated in an acoustically isolated room with dimmed lights in front of a TV monitor surrounded by a uniform luminance field of 5 cd/m2. To obtain a stable pupillary diameter, each subject was adapted to the ambient room light for 10 min before the Visual-evoked potentials (VEPs) recordings. The VEPs were elicited by right monocular stimulation. Visual stimuli consisted of full-field checkerboard patterns (contrast 80%, mean luminance 50 cd/m2) generated on a TV monitor. The reversal rate was 1.55 Hz (3.1 reversal per second). The single checks subtended a visual angle of 15 minutes at a viewing distance of 114 cm while the checkerboard subtended to 23°. Recordings were done with the best corrected visual acuity of > 8/10 at the viewing distance. Subjects were instructed to fixate with their right eye on a red dot in the middle of the screen with the contralateral eye covered by a patch to maintain stable fixation. The VEPs were recorded via the scalp through silver cup electrodes positioned at Oz (active electrode) and at the Fz (reference electrode, 10/20 system). A ground electrode was placed on the right forearm. Signals were amplified by Digitimer™ D360 pre-amplifiers (band-pass 0.05–2000 Hz, gain 1000) and recorded with a CED™ power 1401 device (Cambridge Electronic Design Ltd, Cambridge, UK). A total of 600 consecutive sweeps (each lasting 200 ms) were collected and sampled at 4000 Hz.

After applying a 100 Hz low-pass digital filter off-line, cortical responses were partitioned in six sequential blocks of 100 consisting of at least 95 artefact-free sweeps. Responses in each block were averaged off-line (“block averages”) using the Signal™ software package version 4.10 (CED Ltd).

VEPs components were identified according to their latencies: N1 was the most negative peak between 60 and 90 ms, and P1 was the most positive peak following N1 between 80 and 120 ms. The N2 was the most negative peak following P1 between 125 and 150 ms (Fig. 2). We measured the peak-to-peak amplitude of the N1-P1 and P1-N2 complexes. Habituation was defined as the slope of the linear regression line for the 6 blocks [16].

#### Somatosensory-evoked potentials

Somatosensory evoked potentials (SSEPs) were elicited by electrical stimulation of the right median nerve at the wrist using a constant current square wave pulse (0.1 ms width, cathode positioned proximally) with a stimulus intensity set at 1.5 times the motor threshold; the repetition rate was 4.4 Hz. The 1st active electrode was placed over Erb’s point ipsilateral to the stimulus referenced to the contralateral side. The 2nd and 3rd recording electrodes were positioned over the 5th cervical spinous process (Cv5) and over the contralateral parietal area (C3′, 2 cm posterior to C3 in the international 10–20 system). Both were referenced to the Fz. The ground electrode was on the right arm. The SSEP signals were amplified and recorded with the same hardware/software electronic equipment as described above for VEPs recordings.

The subjects sat relaxed in a comfortable chair in a well-lit room with both eyes open during the recordings. They were asked to fix their attention on the stimulus-induced thumb movement. Five hundred consecutive sweeps of 50 ms, sampled at 5000 Hz, were collected.

After digital filtering of the signal between 0–450 Hz, the various SSEP components (N9, N13, N20, P25 and N33) were identified according to their respective latencies. We measured peak-to-peak amplitudes of the peripheral N9 (recorded under the active Erb’s point electrode), the cervical N13 component (recorded under the active Cv5 electrode), and the cortical N20-P25 and P25-N33 components (recorded under the active C3′ scalp electrode).

Thereafter, since a clear-cut habituation (or its lacking) already from the 2nd block of 100 averaged responses onwards was observed in previous studies by independent groups [17, 18], the first 200 evoked responses were partitioned in two sequential blocks of 100 responses (Fig. 3). Each block was averaged off-line (“block averages”) and analysed for N20-P25 amplitudes. Habituation was expressed as the slope of the linear regression line for the two blocks [18].

For both VEPs and SSEPs, the artefacts were automatically rejected using the Signal™ artefact rejection tool if the signal amplitude exceeded 90% of analogue-to-digital converter (ADC) range. The EP-signal was corrected off-line for DC-drifts.

### Procedure

Somatosensory- and visual-evoked potential recordings were performed in random order during a single session including baseline (time 0) and 1-month after during the ketogenesis as verified through a ketone bodies urinary stick (ketur-test). This was performed on the same day of the recording session. All recordings were collected in the afternoon (between 02.00 and 6.00 p.m.) by the same investigators (D.D.L and C.D.L.) who were not involved in recruitment and inclusion of subjects. These investigators also did not meet the participants prior to the examination. All recordings were numbered anonymously and analysed blindly off-line by one investigator (M.B.), who was not blinded to the order of the blocks.

### Statistical analysis

We used the Statistica for Windows (StatSoft Inc.) version 8.0 for all analyses. A Kolmogorov-Smirnov test showed that VEPs and SSEP components latencies and amplitudes had a normal distribution. A General Linear Model approach was used to analyse the “between-factor” × “within-factors” interaction effect. The between-subject factor was “group” or “time” (before vs. after KD) and within-subject factor was “blocks”. There were two models of repeated measure ANOVA (rm-ANOVA), one for VEPs and another for SSEP as well as univariate ANOVAs to investigate the interaction effect. Univariate results were analysed only if Wilks’ Lambda multivariate significance criterion was achieved. The sphericity of the covariance matrix was verified with the Mauchly Sphericity Test; in the case of violation of the sphericity assumption, the Greenhouse-Geisser (G-G) epsilon (ε) adjustment was used.

Sphericity cannot be evaluated for the second model of ANOVA (SSEP) because there were fewer than three levels of repeated measurement factors. In rm-ANOVA and ANOVA models, partial eta^2^$\left({\eta}_p^2\right)$ and observed power (op) were used as measures of effect size and power, respectively. In a third rm-ANOVA model, we used “type of KD” (normocaloric vs. hypocaloric) as the between-subject factor. The within-subject factor was “time” (before and after KD). These were used to evaluate the relative contribution of diet on neurophysiological results. To define which comparison(s) contributed to the major effects, post hoc tests were performed with a Tukey Honest Significant Difference (HSD) test.

Paired-sample t tests were used to compare the clinical data before vs. after KD. P values less than 0.05 were considered to indicate statistical significance.

## Results

### Clinical characteristics

**Fig. 1:**
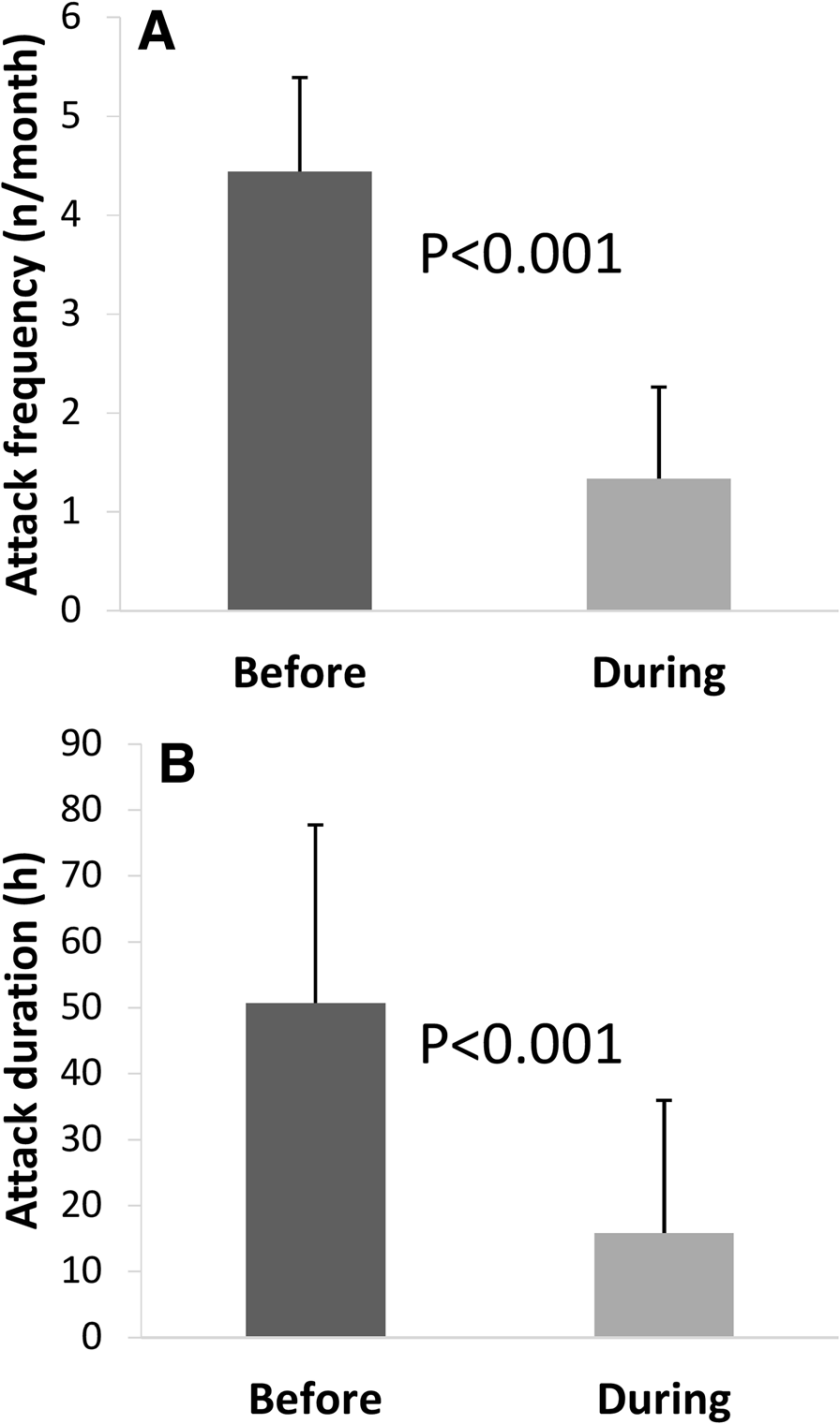
Mean migraine attack frequency [**a**] and duration [**b**] before and during a 1-month ketogenic diet

The clinical characteristics of migraine patients before and after 1-month of KD are displayed in Table 2 and Fig. 1. This figure also shows that there was a significant reduction in attack frequency (from 4.4 ± 2.7 to 1.3 ± 1.1 attacks/month, *t* = 4.70, *p* < 0.001) and attack duration (from 50.7 ± 26.9 to 15.8 ± 20.1 hours, *t* = 5.43, *p* < 0.001) after 1-month duration KD.

**Table 2:** Baseline clinical and demographic characteristics of the total group of migraine patients (M-tot) and its subgroups undergoing normo- (M-norm) or hypo- (M-hypo) caloric ketogenic dietetic regimens

Characteristics	HV (*n* = 18)	M-tot (*n* = 18)		M-norm (*n* = 8)		M-hypo (*n* = 10)	
Women (n)	16	16		7		9	
Age (years)	38.8 ± 9.3	38.8 ± 9.3		36.7 ± 9.3		40.5 ± 9.3	
Duration of migraine history (years)		18.0 ± 9.9		15.1 ± 7.0		20.4 ± 11.7	
		Before	After	Before	After	Before	After
Attack frequency/month (n)		4.4 ± 2.7	1.3 ± 1.1 *	4.2 ± 2.5	1.1 ± 1.4 *	4.6 ± 2.9	1.5 ± 0.7 *
Attack duration (hours)		50.7 ± 26.9	15.8 ± 20.1 *	55.5 ± 24.8	8.7 ± 13.8 *	46.9 ± 29.3	21.5 ± 23.1 *
Body mass index (BMI)	25.1 ± 4.3	25.5 ± 4.6	24.0 ± 1.2 *	21.7 ± 1.8	20.6 ± 1.6 *	28.5 ± 3.7	26.8 ± 3.5 *

Data are expressed as means ± SD. * = p < 0.05 before vs. after 1-month ketogenic diet

### Basic neurophysiological parameters

**Fig. 2:**
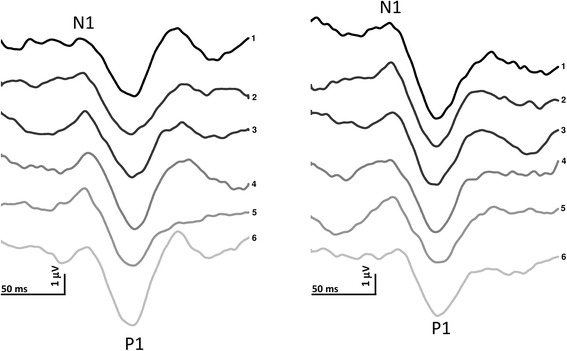
Representative recordings of visual evoked potentials in a migraine patient recorded before (left panel) and after a 1-month (right panel) ketogenic diet

**Fig. 3:**
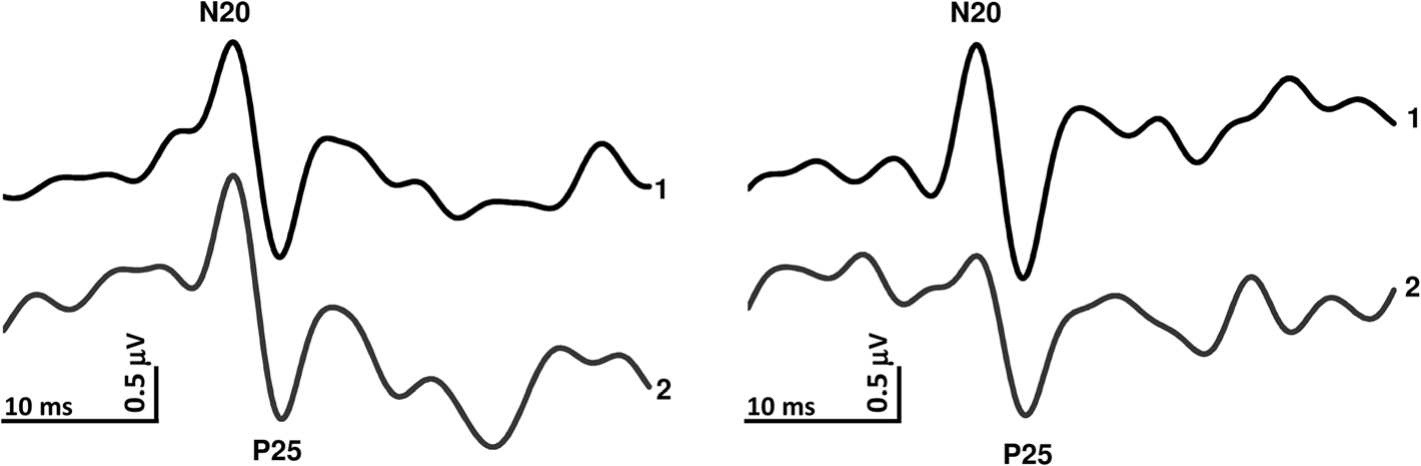
Representative recordings of somatosensory-evoked potentials in a migraine patient recorded before (left panel) and after a 1-month (right panel) ketogenic diet

Assessable VEP and SSEP recordings were obtained from all subjects participating in the study (Figs. 2 and 3). On grand-average the VEP (N1, P1, and N2, Table 3) and SSEP (N9, N13, N20, P25, and N33, Table 4) latencies and their corresponding amplitudes (VEP N1-P1 and P1-N2; SSEP N9, N13, N20-P25, and P25-N33) were not significantly different between migraineurs and healthy volunteers (*P* > 0.05).

**Table 3:** Latencies (in milliseconds) of VEPs in healthy volunteers (HV), in the total group of migraine patients (M-tot) and its subgroups undergoing normo- (M-norm) or hypo- (M-hypo) caloric ketogenic diet (KD) before and after 1-month KD

Electrophysiological parameters	HV (*n* = 18)	M-tot (*n* = 18)		M-norm (n = 8)		M-hypo (n = 10)	
		Before	After	Before	After	Before	After
N1	75.3 ± 7.4	76.4 ± 5.9	76.1 ± 5.5	75.2 ± 3.6	75.3 ± 4.2	77.2 ± 7.2	78.4 ± 6.2
P1	103.7 ± 4.8	102.9 ± 9.0	103.2 ± 9.0	101.8 ± 4.4	100.1 ± 6.6	103.7 ± 7.4	105.4 ± 10.1
N2	145.1 ± 8.3	144.9 ± 9.9	143.8 ± 10.4	141.8 ± 11.6	141.7 ± 11.9	147.2 ± 8.4	145.0 ± 11.3
N1-P1 1^st^ amplitude block (μV)	8.3 ± 2.3	8.5 ± 4.0	9.3 ± 3.9	10.2 ± 3.7	10.5 ± 3.8	7.0 ± 3.8	8.4 ± 4.0
N1-P1 amplitude slope	- 0.3 ± 0.2	0.1 ± 0.3 §	- 0.2 ± 0.3 *	0.2 ± 2.6	- 0.2 ± 0.3 *	0.05 ± 0.3	- 0.2 ± 0.3 *
P1-N2 1^st^ amplitude block (μV)	7.6 ± 3.3	9.1 ± 4.2	8.3 ± 4.4	9.7 ± 4.5	9.3 ± 5.0	8.6 ± 4.1	7.5 ± 3.9
P1-N2 amplitude slope	−0.3 ± 0.7	- 0.1 ± 0.2	- 0.05 ± 0.3	0.005 ± 0.2	- 0.02 ± 0.2	- 0.1 ± 0.3	- 0.06 ± 0.1

Data are expressed as means ± SD. * = *p* < 0.05 before vs. after 1-month KD. § = p < 0.01 vs. HV

**Table 4:** Grand-average somatosensory evoked potentials (SSEPs) latencies and amplitudes in healthy volunteers (HV), in the total group of migraine patients (M-tot) and its subgroups undergoing normo- (M-norm) or hypo- (M-hypo) caloric ketogenic diet (KD) before and after 1-month KD

Electrophysiological parameters	HV (*n* = 18)	M-tot (*n* = 18)		M-norm (*n* = 8)		M-hypo (n = 10)	
		Before	After	Before	After	Before	After
N9 (ms)	9.7 ± 0.7	9.8 ± 0.9	9.8 ± 0.7	9.5 ± 0.7	9.5 ± 0.7	10.0 ± 1.0	10.1 ± 0.5
N13 (ms)	13.4 ± 0.8	13.4 ± 1.0	13.3 ± 1.0	13.0 ± 0.7	13.0 ± 0.9	13.6 ± 1.1	13.6 ± 1.0
N20 (ms)	19.4 ± 1.2	19.1 ± 1.3	19.1 ± 1.2	18.4 ± 0.7	18.7 ± 0.8	19.7 ± 1.4	19.5 ± 1.4
P25 (ms)	24.9 ± 2.1	24.9 ± 3.1	25.7 ± 3.1	24.3 ± 2.2	25.1 ± 2.3	25.4 ± 3.7	26.1 ± 3.7
N33 (ms)	30.6 ± 2.4	31.5 ± 2.3	31.4 ± 3.1	31.2 ± 1.9	31.3 ± 3.1	31.8 ± 2.6	31.5 ± 3.3
N9-p (μV)	2.9 ± 1.4	3.0 ± 1.8	3.1 ± 2.0	3.3 ± 2.3	3.7 ± 2.3	2.7 ± 1.4	2.6 ± 1.6
N13-p (μV)	1.6 ± 0.5	1.6 ± 0.5	1.7 ± 0.5	1.8 ± 0.6	2.1 ± 0.4	1.4 ± 0.5	1.4 ± 0.4
N20-P25 (μV)	1.8 ± 0.6	1.8 ± 0.7	1.8 ± 0.9	1.8 ± 0.8	1.9 ± 1.1	1.8 ± 0.5	1.7 ± 0.6
P25-N33 (μV)	0.8 ± 0.4	0.9 ± 0.5	0.9 ± 0.4	1.0 ± 0.7	0.9 ± 0.2	0.9 ± 0.3	1.0 ± 0.5
N20-P25 1^st^ amplitude (μV)	2.3 ± 0.9	2.1 ± 0.9	2.1 ± 1.2	2.2 ± 1.2	2.6 ± 1.5	2.0 ± 0.6	1.7 ± 0.8
N20-P25 amplitude slope	- 0.3 ± 0.7	0.3 ± 0.7 §	- 0.5 ± 0.6 *	0.4 ± 0.5	- 0.7 ± 0.7 *	0.2 ± 0.9	- 0.3 ± 0.4 *

Data are expressed as means ± SD. * = p < 0.01 paired sample *t* test before vs. after 1-month ketogenic diet. § = p < 0.01 ANOVA vs. HV

Before KD short-term intervention, the migraine patients lacked habituation in response to both visual and somatosensory repetitive stimulations. In fact, in the rm-ANOVA model with VEP N1-P1 or SSEP N20-P25 peak-peak amplitudes as dependent variable, multivariate test was significant for the “group” × “blocks” interaction effect (Wilks’ Lambda = 0.711, F_5,30_ = 5.395, *p* = 0.001 for VEP; Wilks’ Lambda = 0.838, F_5,30_ = 6.593, *p* = 0.015 for SSEP). The linear regression slope of VEP (N1-P1) and SSEP (N20-P25) amplitudes over all blocks differed significantly between the two groups (F_1,34_ = 27.029, *p* < 0.0001 for VEP; F_1,34_ = 6.613, *p* = 0.015 for SSEP). By contrast, in the rm-ANOVA model with VEP P1-N2 peak-peak amplitude as dependent variable, multivariate test was not significant for the “group” × “blocks” interaction effect (Wilks’ Lambda = 0.918, F_5,30_ = 0.538, *p* = 0.746).

### Dietetic intervention effects

On grand-average the VEP (N1, P1, and N2, Table 3) and SSEP (N9, N13, N20, P25, and N33, Table 4) latencies and their corresponding amplitudes (VEP N1-P1 and P1-N2; SSEP N9, N13, N20-P25, and P25-N33) were not significantly different before and after KD in migraineurs overall and in both KD regimen groups (*P* > 0.05).

**Fig. 4:**
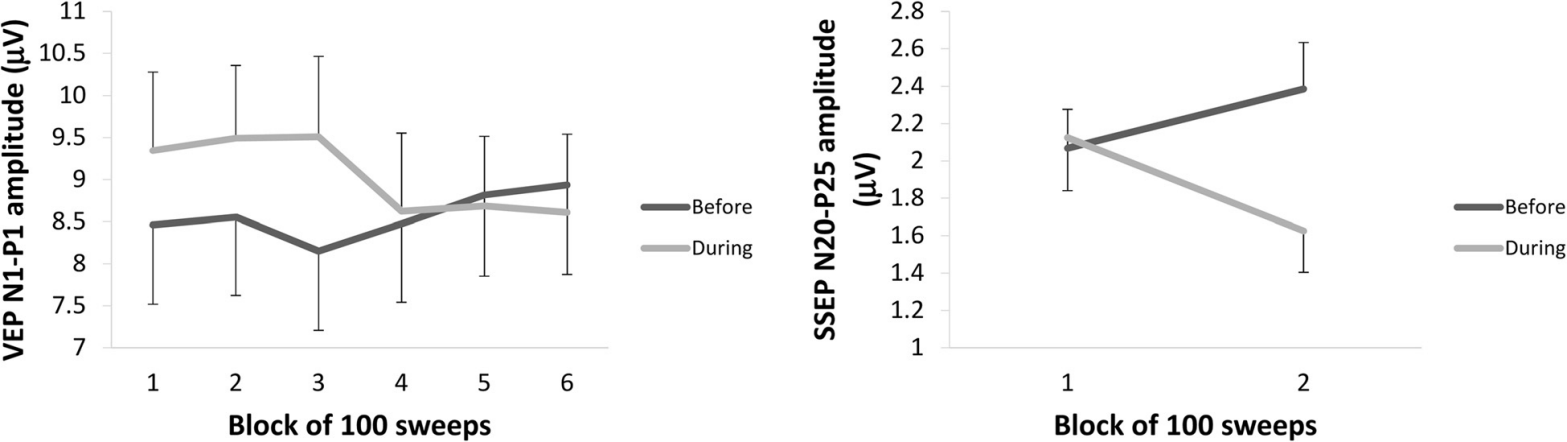
Raw amplitudes (mean ± SEM) of visual evoked potential N1-P1 (left graph) and somatosensory evoked potential N20-P25 (right graph), respectively, in 6 and 2 sequential blocks of 100 recordings

In the rm-ANOVA model using VEP N1-P1 peak-peak block amplitude as dependent variable, the multivariate test was significant for the “time” × “blocks” interaction effect (Wilks’ Lambda = 0.631, F_5,30_ = 3.27, *p* = 0.019). The univariate rm-ANOVAs for N1-P1 peak-peak amplitudes confirmed the significant interaction factor effect (F_5,170_ = 2.71, *ε* = 0.79, *p* = 0.033, partial η2 = 0.078, op = 0.81) observed at the multivariate test. The linear regression N1-P1 slope of VEP amplitudes over all blocks differed significantly between before and after KD (F_1,34_ = 8.92, *p* = 0.005, partial η2 = 0.208, op = 0.83; raw data are shown in Fig. 4).

In the rm-ANOVA model using VEP P1-N2 peak-peak block amplitude as dependent variable, multivariate test was not significant for the “time” × “blocks” interaction effect (Wilks’ Lambda = 0.971, F_5,30_ = 0.180, *p* = 0.968).

In the rm-ANOVA model using SSEP N20-P25 peak-peak block amplitude as the dependent variable, the multivariate test was significant for the “time” × “blocks” interaction effect (Wilks’ Lambda = 0.711, F_5,30_ = 13.83, *p* = 0.0007). The univariate rm-ANOVAs for N20-P25 peak-peak amplitude confirmed the significant interaction factor effect (F_1,34_ = 13.83, *p* = 0.0007, partial η2 = 0.289, op = 0.95) observed at the multivariate test. The linear regression N20-P25 slope of SSEP amplitudes over the two blocks differed significantly between the before and after KD (F_1,34_ = 13.83, *p* = 0.0007, partial η2 = 0.289, op = 0.95; raw data are shown in Fig. 4).

In the rm-ANOVA model with VEP N1-P1 or SSEP N20-P25 amplitude slope (before or after KD) as the dependent variable, the multivariate test was not significant for the “time” × “type of KD” interaction effect (Wilks’ Lambda = 0.711, F_1,16_ = 1.13, *p* = 0.304; Wilks’ Lambda = 0.881, F_1,16_ = 2.17, *p* = 0.160, respectively). Nevertheless, in both KD regimen groups (normo- and hypo- caloric), the baseline attack frequency, attack duration, and BMI were significantly reduced after a month of diet (Table 2).

## Discussion

This study confirms our previous clinical finding that migraine features significantly improve in terms of attack frequency and duration during a month of ketogenic diet [8, 9]. Moreover, this study also confirms that before KD short-term interventions, the migraine patients lacked habituation in response to visual (VEP N1-P1 slope: + 0.09) and somatosensory (SSEP N20-P25 slope: + 0.32) repetitive stimulations [19, 20].

The most striking finding is that KD could significantly normalize the habituation deficit on evoked potentials as long as migraine clinical features improved. This was a general behavioural cortical process in response to KD because it was independent from the stimulus modality inducing the lack of habituatory effect. Moreover, our neurophysiological findings were independent from loss of weight because both patients who followed normo-caloric and those who followed hypo-caloric regimen habituate normally during KD.

Various mechanisms might underlie these findings. Most of these are borrowed from the clinical and experimental studies performed in epilepsy. These include induction of neural plasticity, changes in cortical excitability and enhancement in energetic metabolism.

Habituation, i.e. the “response decrement as a result of repeated stimulation” is a basic form of learning and memory [21]. Such changes in behavioural response offers insight into the adaptive short- and long- term plasticity mechanisms that correspond to changes in neuronal excitability. These include a decrease in evoked potential amplitudes that further depend on the underlying genetic load [22–24].

Interestingly, in animal models, KD seems to precisely affect synaptic plasticity and cortical excitability. In freely behavioural rats, a ketogenic diet—although not significantly altering baseline excitability—did reduce the magnitude of long-term potentiation plastic mechanisms in the hippocampal dentate gyrus [11, 25]. Our results seem to be in perfect agreement with those provided by the animal models. While we did not find significant differences in term of initial baseline excitability as reflected in the non-significant changes in 1^st^ block VEP and SSEP amplitudes, we did observe that KD reduced the interictal-evoked potential amplitude potentiation and normalized the response. This is not unusual. Indeed, in a habituation paradigm, early and late responses may behave differently because they are regulated by different mechanisms. In fact, according to the dual-process theory, facilitation/sensitization (increasing response) competes with habituation (decreasing response) to determine the final behavioural outcome. Facilitation occurs at the beginning of the stimulus session and accounts for the initial transitory increase in response amplitude. Habituation occurs throughout the recording session and accounts for the delayed response decrease [26]. Therefore, our results suggest that KD selectively affects delayed habituation by reducing migraineurs’ cortical hyperresponsivity. This avoids any influence on the initial facilitatory mechanism.

Reduced potentiation/hyperresponsivity mechanisms in ketogenic diet-fed rats [11, 25] is consistent with a general modulation of cortical excitability. This was confirmed by the observation that KD is a useful tool in controlling childhood seizures [27, 28]. In hippocampal in vitro seizures models, KD feeding limited epileptic activity in several portions of the hippocampus by promoting ATP-sensitive K+ channel activity that is implicated in plasma membrane excitability and in regulation of aerobic metabolism [29–31]. Both augmentation of fast GABAergic (GABA_A_) neurotransmission and decreased probability of excitatory neurotransmitter release from presynaptic neurons have been implicated in the anticonvulsivant actions of the KD [32, 33]. In fact, elevated concentrations of KBs have been reported to increase and maintain synaptosomal GABA contents at a higher value [34] and increase the intracellular concentration [35] and cause presynaptic input reduction of glutamate [31]. In healthy humans, KD is associated with a significant enhancement of intracortical inhibition as measured with transcranial magnetic stimulation [10]. These experimental observations are of particular interest in migraine because we have previously reported that the migraineurs’ brains are characterized by an imbalance between excitation and inhibition in the sensory cortices [36]. In particular, distinct cortical inhibitory mechanism, the so-called lateral inhibition, were reported to be impaired interictally in the visual [20] and somatosensory [37] cortices of migraine patients. This impairment may contribute to the interictal propensity to lack EP amplitude habituation [20]. Further evidence for defective inhibitory mechanisms in the visual cortex of migraine with and without aura patients came also from studies using paired-pulse suppression of visual evoked potentials [38, 39]. We hypothesize that KD works by heightening GABAergic and diminishing excitatory activities. This may have forced the delayed reduction of VEP and SSEP amplitudes and restored normal habituatory behaviour.

Another possible mechanism of KD is the restoration of normal EP habituation via potentiation of mitochondrial energy metabolism. The KD may induce a strengthened mitochondrial metabolism and biogenesis [40] and improve energy production throughout the oxidative respiratory complex [41]. This leads to a more efficient synaptic transmission and plasticity [42, 43]. This is of obvious relevance because neuronal excitability depends on energy metabolism and because convergent data from various laboratories have shown that brain oxidative metabolism, mitochondrial functioning, and energetic production are significantly reduced in the brain of migraineurs between attacks [44–46].

This mitochondrial dysfunction in migraine causes anaerobic metabolites (e.g. lactate) to increase in the brain. In healthy humans during sustained visual stimulation, the increasing habituation of evoked potentials coincides with a decrease in cortical lactate levels [47]. Migraineurs show the opposite effect during continuous visual stimulation [45]. This is of interest because the elevation of cerebral lactate concentration is a common finding in patients with mitochondrial dysfunction [48]. Consequently, we speculate that the KD regimen may dampen the excessive brain lactate accumulation produced during sensory habituation deficit because KD improves mitochondrial metabolism. This favours the restoration of a normal reducing response, i.e. habituation.

Finally, we have shown that VEP and SSEP amplitudes habituate normally following KD irrespective of the type of KD (normo-caloric or hypo-caloric). Because the different diets were assigned according to the baseline weight, this implies that neither the pre-diet BMI nor the amount of lipids and calories related to the diet seem to influence the outcome of neurophysiological measures. Moreover, baseline clinical and neurophysiological variables were not significantly different between subgroups (see Tables 2 and 3). Therefore, these observations favour the hypothesis that the switch from glucose to KBs as an energetic substrate in patients’ brain could play a relevant role in changing their interictal abnormal information processing.

As with all studies, our findings need to be considered with our study limitations. First, the number of patients and the period of observation are too small to generalize our results, although our cohort was sufficient to disclose the same behavioural phenomenon in response to KD using two different stimulus modality. The issue of low number of patients is particularly true for the analysis of the subgroups of patients who underwent normo- or hypo-caloric ketogenic dietetic regimens. Future studies should attempt to clarify the possible cortical effects of different ketogenic dietetic regimens in a larger cohort of patients. Moreover, we acknowledge as a limitation of the present study the lack of inclusion of a patients control group staying on the waiting-list not undergoing the dietetic intervention in order to control for effects of repeated measurements at the neurophysiological level and for unpredictable spontaneous clinical improvement. However, this point should not consider to be detrimental since a recent study in migraine proved that, at least for the visual modality, evoked potential amplitude habituation has a sufficient repeatability over an average test-retest time interval of 60 days [49], and because we previously proved that only 1-month KD, but not a standard diet, was clinically effective in a population of migraine patients [9].

Another shortcoming of our study is that we cannot exclude the possibility that other mechanisms not related to the ketogenesis could account for the results we have observed. For instance, other possibilities are that KD induces effects by promoting hypoglycaemia, lack of fluctuations of insulin blood levels, or by lowering the activity of the so-called nutrient-integrating pathways that sense and respond to fluctuations in nutrient levels [50].

## Conclusions

In conclusion, our study proves that in migraineurs KBs has central nervous system effects in parallel with clinical improvement. In our migraine patients who were characterized by a baseline increasing of VEP and SSEP amplitude responses during repetitive stimulation (deficit of habituation), this dietetic intervention was followed by a significant EPs amplitude decrement (habituation).

We have hypothesized that several interacting mechanisms may be at work in the clinical and neurophysiological actions of the KD. Future works will clarify whether cerebral cortex is the primary site of KD-related changes or if the latter are the expression of its ability to modulate subcortical structures including the brainstem and thalamus. In turn, these may cause abnormal cortical activation.

## Abbreviations

BMI, body mass index; HSD, tukey honest significant difference; KD, ketogenic diet; MAD, atkins modified diet; rm-ANOVA, repeated measure ANOVA; SSEPs, somatosensory evoked potentials; VEPs, visual evoked potentials; VLCKD, very low-calorie ketogenic diet
